# Adverse maternal and neonatal outcomes among singleton pregnancies in women of very advanced maternal age: a retrospective cohort study

**DOI:** 10.1186/s12884-018-2147-9

**Published:** 2019-01-03

**Authors:** Yuelin Wu, Yan Chen, Minxue Shen, Yanfang Guo, Shi Wu Wen, Andrea Lanes, Ruth Rennicks White, Adewumi Adanlawo, Mark Walker, Xiaolin Hua

**Affiliations:** 1 Department of Obstetrics and Gynecology, Xinhua Hospital, Shanghai Jiao Tong University School of Medicine, 1665 Kongjiang Road, Shanghai, 200092 China; 2 OMNI Research Group, Clinical Epidemiology Program, Ottawa Hospital Research Institute, Ottawa, Ontario Canada; 3 Department of Dermatology, Xiangya Hospital, Central South University, Changsha, Hunan China; 4 Better Outcomes Registry & Network Ontario, Ottawa, Ontario Canada; 5 Children’s Hospital of Eastern Ontario Research Institute, Ottawa, Ontario Canada; 6 School of Epidemiology and Public Health, University of Ottawa, Ottawa, Ontario Canada; 7 Maternal-Fetal Medicine, Regina General Hospital, University of Saskatchewan, Reginal, Saskatchewan, Canada; 8 Department of Obstetrics and Gynecology, Faculty of Medicine, OMNI Research Group, University of Ottawa, 501 Smyth Road, Ottawa, Ontario K1H8L6 Canada

**Keywords:** Very advanced maternal age, Maternal and neonatal pregnancy outcomes, Ischemic placental diseases, Assisted reproductive technology

## Abstract

**Background:**

There is an increasing prevalence of women who tend to delay childbirth until a very advanced age. However, there is sparse data regarding very advanced maternal age (vAMA) and the interplay between vAMA and assisted reproductive technology (ART) on adverse perinatal outcomes. The study aimed to assess the risk of adverse maternal and neonatal outcomes of vAMA women (≥43 years), and to investigate the effect of maternal age on adverse maternal and neonatal outcomes in ART pregnancies.

**Methods:**

Data was obtained from a population-based retrospective cohort of women who delivered in Ontario, Canada, between April 1st, 2012 and March 31st, 2015. The adjusted relative risks (ARR) and 95% confidence intervals (CI) for adverse maternal and neonatal outcomes were estimated by using multivariate log-binomial regression models among age groups. All models were stratified by the utilization of ART (ART and spontaneous conceptions).

**Results:**

Women at vAMA had a higher risk of composite outcome comprised of preeclampsia, intrauterine growth retardation, stillbirth, and placental abruption than the younger counterparts (ARR = 1.38, 95% CI: 1.23–1.55 compared to mothers aged 20–34; ARR = 1.26, 95% CI: 1.12–1.42 compared to mothers aged 35–42). Increased risk of the primary outcome in ART compared to spontaneous conception was only observed in women aged 20–34 years (ARR = 1.24, 95% CI: 1.14–1.35). For women conceived with ART, the risk for the primary outcome significantly increased in women at vAMA (ARR = 1.29, 95% CI: 1.01–1.65 compared to mothers aged 20–34; ARR = 1.36, 95% CI: 1.06–1.74 compared to mothers aged 35–42).

**Conclusion:**

Women at vAMA have higher risks of adverse maternal and neonatal outcomes. Although the utilization of ART may carry an independent role for adverse perinatal outcomes, it does not further enhance the adverse effect of vAMA.

**Electronic supplementary material:**

The online version of this article (10.1186/s12884-018-2147-9) contains supplementary material, which is available to authorized users.

## Background

Advanced maternal age (AMA) is defined as women aged 35 years or greater at the estimated date of delivery [1–3]. Recently, the prevalence of AMA has increased, and some women are even delaying childbirth until their forties [4–6]. In Canada, the number of live births in women aged above 35 increased from 59,755 in 2005 to 78,615 in 2014, and the number of births in women aged above 40 tripled from 2005 to 2014 [7, 8].

Studies have found women of AMA to be at an increased risk for obstetric complications and adverse perinatal outcomes, including gestational diabetes mellitus (GDM), hypertensive disorders, preeclampsia, perinatal birth defects, stillbirth and preterm birth [9, 10]. With progression in advanced technologies in perinatology, pregnancy outcomes in women of advanced age has been improved [11–13]. Jackson et al. suggested that the period of obstetric risk should be considered to be postponed after age 40 years or even age 45 years [1]. Studies also suggested that women with very advanced maternal age (vAMA), defined as 45 or older, were at higher risk of adverse pregnancy outcomes than women with AMA [4, 10, 14–16]. However, some studies concluded that there is no definite medical reason for excluding vAMA women from attempting pregnancy on the basis of age alone [17, 18]. Currently, there was no consensus as to the degree of association or to the impact of maternal age.

In addition, although fertility declines with age, assisted reproductive technology (ART) have given a larger proportion of vAMA women the opportunity to become pregnant. However, ART has been considered as a risk factor for adverse pregnancy outcomes compared with spontaneous conceptions (SC) [1, 18]. Little is known about the interplay between age-related and ART-related risks [1, 19]. The aim of this study was to assess the risk of maternal and neonatal outcomes of women with vAMA, and to assess the interaction effects of maternal age and ART pregnancies on perinatal outcomes.

## Methods

### Study design and data source

In this population-based retrospective cohort study, we used data from Better Outcomes Registry & Network (BORN) Ontario in Canada. BORN Ontario is a provincial prescribed birth registry under the Personal Health Information Protection Act, 2004 (PHIPA). As a registry, BORN is afforded the authority to collect, use and disclose personal health information without consent for the purpose of facilitating or improving the provision of healthcare. All requests made to BORN Ontario for data access will be managed in accordance with the privacy legislation (PHIPA) [20]. The BORN data contains maternal demographics, health behaviors and reproductive history, as well as clinical information related to pregnancy, labor, birth and fetal and neonatal outcomes. Social economic status including neighbourhood household income and education quintiles were obtained from linked 2011 Canadian Census data by maternal residence postal code.

### Study population

Singleton pregnancies among women aged ≥20 years who delivered at 20 weeks of gestation or greater or birth weight larger than 500 g between April 1st, 2012 and March 31st, 2015 were included in this study.

### Exposure measurement

Mother’s age at delivery was our independent variable of interest. Maternal age was recorded as continuous variable in BORN data. We categorized them into three groups: 20–34 years, 35–42 years and ≥ 43 years. vAMA was defined as women aged ≥43 years at delivery. This cutoff was chosen owing to the limited number of women above 45 as well as literature support [2, 21].

### Main covariate

Type of conception was the main covariate. Type of conception was classified as ART and SC. ART conceptions included: intrauterine insemination (IUI); IUI with ovulation induction but without in-vitro fertilization (IVF); IVF; IVF with intracytoplasmic sperm injection (ICSI); ovulation induction without IVF (i.e. clomid or follicle-stimulating hormone); and vaginal insemination. Pregnancies recorded with SC were categorized as SC group.

### Other covariates

We included a wide range of potential confounders for adverse maternal and neonatal outcomes: parity (0, 1, ≥2), neighbourhood household median income quintile (lowest, 2nd, 3rd, 4th, highest), neighbourhood education quintile (percentage of adults 26 to 64 years having a university degree, [lowest, 2nd, 3rd, 4th, highest]), pre-pregnancy body mass index (BMI) categories (underweight, normal, overweight or obese), drug/alcohol/tobacco use (yes or no), maternal pre-existing health problems (pre-existing hypertension, pre-existing diabetes, heart disease, pulmonary disease, endocrine disorder or thrombophilia [yes or no]), GDM (yes or no), preeclampsia (yes or no).

### Outcome

The primary outcome was a composite of preeclampsia, intrauterine growth retardation (IUGR), placental abruption and stillbirth, also known as ischemic placental diseases [22]. Placental ischemia is a consequence of impaired trophoblast invasion and too shallow spiral artery conversion that may develop preeclampsia, IUGR, placental abruption and part of stillbirth [23].

The secondary outcomes covered a series of maternal and neonatal outcomes. Maternal complications included preterm birth, GDM, placental previa, postpartum hemorrhage (PPH), maternal intensive care unit (ICU) admission, and maternal mortality related to pregnancy and birth. Neonatal outcomes included small for gestational age (SGA) < 5th percentile, neonatal death, sentinel congenital anomalies, neonatal intensive care unit (NICU) admission, 5 min APGAR score ≤ 3.

Preeclampsia was defined as preeclampsia or HELLP or eclampsia. IUGR was defined as SGA <10th percentile. Preterm birth was defined as gestational age at delivery < 37 weeks. Sentinel congenital anomalies were listed in Additional file 1: S1.

### Statistical analysis

Maternal demographic characteristics and clinical factors were compared among the three age groups. Continuous variables were described by mean ± standard deviation (SD) or median (interquartile range, IQR). Categorical variables were described by counts and percentages (%). Analysis of variance or Kruskal-Wallis H tests were performed for continuous data, and chi-square tests or Fisher’s exact tests were performed for categorical data.

The incidences of adverse maternal and neonatal outcomes were examined among three age groups. Multivariate log-binomial regression models were used to estimate the adjusted relative risk (ARR) and 95% confidence intervals (CI) of adverse maternal and neonatal outcomes across age groups. Regression model for maternal outcomes were adjusted for parity, neighborhood income, educational level, pre-pregnancy BMI, drug/alcohol/tobacco use, type of conception, and pre-existing health problems. Neonatal outcomes models were further adjusted for GDM and preeclampsia in addition to the aforementioned factors. Interaction effects between maternal age and type of conception and other covariates on adverse maternal and neonatal outcomes were also tested.

All analyses were performed using the Statistical Analysis System (SAS) for Windows, version 9.4 (SAS Institute, Cary, NC), with two-tailed tests and a significance level of *P* < 0.05.

## Results

**Fig. 1:**
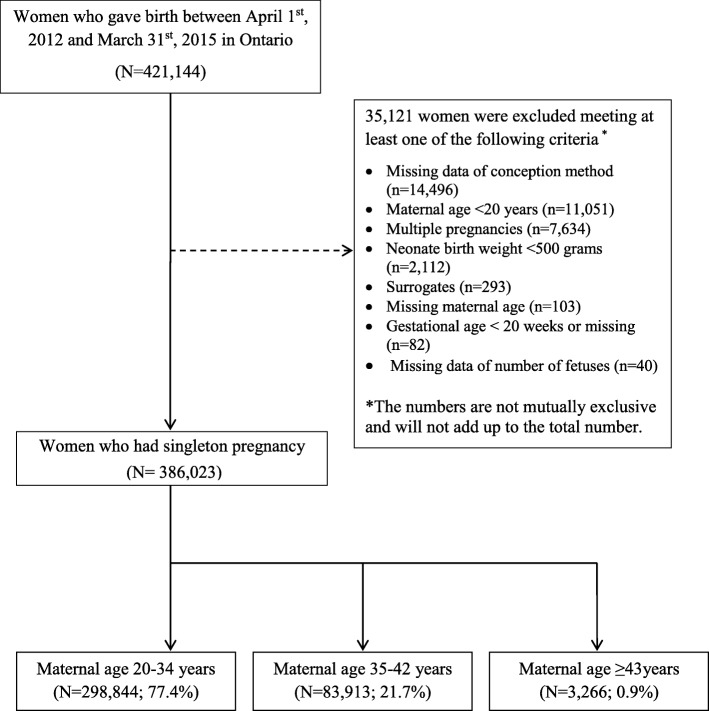
Study flow chart

A total of 421,144 women gave birth in Ontario, Canada between April 1st, 2012 and March 31st, 2015 and 386,023 women met the study inclusion criteria for analysis. Of these, 77.4% were 20–34 years, 21.7% were 35–42 years, and 0.9% were ≥ 43 years age (Fig. 1).

The demographic and clinical characteristics of the participants are shown in Table 1. Gravidity, parity, and pre-pregnancy BMI increased with elevated maternal age. Older women had higher educational and income levels compared to younger women (*P* < 0.001). ART was more common in women of vAMA, with 2.1, 5.9 and 20.1% in women aged 20–34, 35–42 and ≥ 43 years, respectively. There was a significantly higher prevalence of pre-existing hypertension, pre-pregnancy diabetes mellitus, maternal heart disease and endocrine disorders with increased age (*P* < 0.001), while pulmonary disease was significantly more common in women aged 20–34 years. For cases where information was available, drug and tobacco were relatively rare among the older. Alcohol use during pregnancy and gender of the babies were not significantly different.

**Table 1:** Comparison of demographic and clinical characteristics of participants across maternal age groups

	20–34 years		35–42 years		≥ 43 years		Overall	*P*
	n	%	n	%	n	%	n	
N	298,844	77.4	83,913	21.7	3266	0.8	386,023	
Maternal age, year (mean ± SD)	28.7 ± 3.7		37.2 ± 2.0		44.2 ± 1.9			< 0.001
Gravidity (median, IQR)	2 (2)		2 (2)		3 (3)			< 0.001
Parity (Median, IQR)	1 (1)		1 (2)		1 (2)			< 0.001
0	137,021	45.9	22,231	26.5	910	27.9	160,162	< 0.001
1	103,802	34.7	32,606	38.9	958	29.3	137,366	
≥2	53,739	18.0	27,619	32.9	1342	41.1	82,700	
Missing	4282	1.4	1457	1.7	56	1.7	5795	
Neighbourhood income quintile								
1st (Lowest)	69,635	23.3	16,583	19.8	753	23.1	86,971	< 0.001
2nd	55,635	18.6	13,656	16.3	523	16.0	69,814	
3rd	53,391	17.9	13,787	16.4	536	16.4	67,714	
4th	54,983	18.4	16,644	19.8	576	17.6	72,203	
5th (Highest)	52,827	17.7	20,171	24.0	758	23.2	73,756	
Missing	12,373	4.1	3072	3.7	120	3.7	15,565	
Neighbourhood education quintile ^a^								
1st (Lowest)	56,392	18.9	9173	10.9	381	11.7	65,946	< 0.001
2nd	62,349	20.9	13,141	15.7	510	15.6	76,000	
3rd	61,921	20.7	16,257	19.4	574	17.6	78,752	
4th	61,929	20.7	21,041	25.1	767	23.5	83,737	
5th (Highest)	46,246	15.5	21,776	26.0	935	28.6	68,957	
Missing	10,007	3.3	2525	3.0	99	3.0	12,631	
BMI (kg/m^2^) (mean ± SD)	25.3 ± 6.2		25.9 ± 6.1		26.4 ± 6.2			< 0.001
Underweight (<18.5)	15,876	5.3	2634	3.1	64	2.0	18,574	< 0.001
Normal weight (18.5–24.9)	134,814	45.1	35,362	42.1	1235	37.8	171,411	
Overweight (25–29.9)	62,058	20.8	19,415	23.1	780	23.9	82,253	
Obese (≥ 30)	46,129	15.4	14,087	16.8	580	17.8	60,796	
Missing	39,967	13.4	12,415	14.8	607	18.6	52,989	
ART type								
IVF/ IVF + ICSI	2530	0.8	2890	3.4	575	17.6	5995	< 0.001
IUI	3561	1.2	2026	2.4	82	2.5	5669	
Other	87	0.0	72	0.1	< 6	S	160	
None	292,666	97.9	78,925	94.1	2608	79.9	374,199	
Previous cesarean section (yes)	35,687	11.9	18,038	21.5	732	22.4	54,457	< 0.001
Maternal health problems (yes)	30,889	10.3	11,607	13.8	562	17.2	43,058	< 0.001
Pre-existing hypertension	2025	0.7	1369	1.6	117	3.6	3511	< 0.001
Pre-gestational diabetes mellitus	2427	0.8	1216	1.4	76	2.3	3719	< 0.001
Maternal heart disease	3407	1.1	1125	1.3	46	1.4	4578	< 0.001
Maternal pulmonary diseases	12,188	4.1	3038	3.6	127	3.9	15,353	< 0.001
Maternal endocrine disorders	12,569	4.2	5712	6.8	268	8.2	18,549	< 0.001
Thrombophilia	198	0.1	69	0.1	7	0.2	274	0.003
Drug/alcohol/tobacco use (yes)	38,228	12.8	5897	7.0	209	6.4	44,334	< 0.001
Drug use (any drug)	6051	2.0	680	0.8	21	0.6	6752	< 0.001
Alcohol use ^b^	5489	1.8	1499	1.8	65	2.0	7053	0.26
Maternal smoking ^c^	32,974	11.0	4427	5.3	157	4.8	37,558	< 0.001
Infant gender								
Male	153,326	51.3	42,894	51.1	1643	50.3	197,863	0.62
Female	145,323	48.6	40,968	48.8	1622	49.7	187,913	
Undetermined or missing	195	0.1	51	0.1	< 6	S	247	

*ART* assisted reproductive technology, *BMI* body mass index, *ICSI* intracytoplasmic sperm injection, *IQR* interquartile range, *IUI* intra-uterine insemination, *IVF* in vitro fertilization, *S* suppression due to cell < 6, *SD* standard deviation

^a^Percentage of university degrees among population between 25 and 64 years old at dissemination areas level

^b^Alcoholic drink during pregnancy

^c^Smoking at any time during pregnancy

The incidences of the adverse pregnancy outcomes are presented in Table 2 (three age groups) and Additional file 2: Figure S1 (continuous maternal age). The incidence of the primary outcome was 10.41% in women under 35 and 13.35% in women of vAMA. The incidences of the outcomes stratified by the method of conception are shown in Additional file 3: Table S1. Generally, women at vAMA had higher incidence of adverse outcomes regardless of the method of conception.

**Table 2:** Incidence of adverse maternal and neonatal outcomes among maternal age groups

Outcome	20–34 years	35–42 years	≥ 43 years
	n (%)	n (%)	n (%)
Composite outcome (preeclampsia, IUGR, placental abruption and stillbirth)	31,102 (10.41)	8463 (10.09)	436 (13.35)
Preeclampsia	2215 (0.74)	699 (0.83)	52 (1.59)
IUGR	27,616 (9.24)	7314 (8.72)	358 (10.96)
Placental abruption	1330 (0.45)	495 (0.59)	30 (0.92)
Stillbirth	822 (0.28)	290 (0.35)	22 (0.67)
Preterm birth	17,199 (5.76)	5870 (7)	315 (9.64)
Gestational diabetes mellitus	13,618 (4.56)	7393 (8.81)	456 (13.96)
Placental previa	1667 (0.56)	940 (1.12)	55 (1.68)
Postpartum hemorrhage	7533 (2.52)	1836 (2.19)	76 (2.33)
Maternal ICU admission	94 (0.03)	42 (0.05)	< 6
Maternal death related to pregnancy and birth	< 6	<6	< 6
SGA<5th	12,736 (4.26)	3440 (4.10)	169 (5.17)
Neonatal death	411 (0.14)	115 (0.14)	12 (0.37)
Sentinel Congenital Anomalies	1005 (0.34)	360 (0.43)	39 (1.19)
NICU admission	35,096 (11.74)	10,423 (12.42)	518 (15.86)
5 min Apgar score≤3	3100 (1.04)	1008 (1.2)	60 (1.84)

*ICU* intensive care unit, *IUGR* intrauterine growth retardation, *NICU* neonatal intensive care unit, *SGA* small for gestational age

**Fig. 2:**
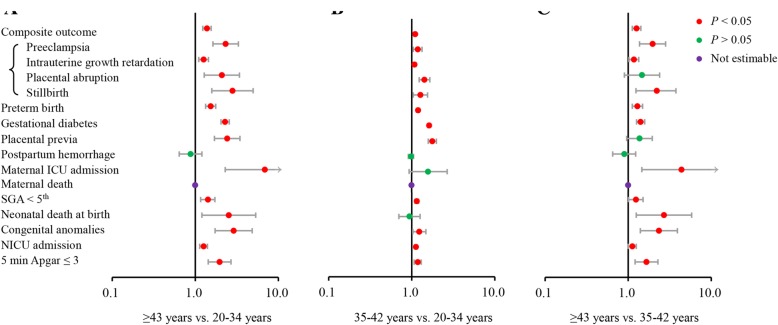
Effect size of advanced and very advanced maternal age on adverse maternal and neonatal outcomes. The composite outcome includes preeclampsia, intrauterine growth retardation, placental abruption and stillbirth. Models for maternal outcomes were adjusted for parity, neighborhood income, educational level, pre-pregnancy body mass index, drug/alcohol/tobacco use, type of conception, maternal pre-existing health problems (preexisting hypertension, pre-existing diabetes mellitus, maternal heart disease, maternal pulmonary diseases, maternal endocrine disorders, hematologic disorders). Models for neonatal outcomes were adjusted for parity, neighborhood income, educational level, pre-pregnancy body mass index, drug/alcohol/tobacco use, type of conception, maternal pre-existing health problems, gestational diabetes mellitus, and preeclampsia. Dots signify relative risks, and bars signify 95% confidence intervals. Red dots signify significant relative risks, green dots signify insignificant results, and purple dots signify non-estimable results

The adjusted RRs of AMA and vAMA for maternal and neonatal outcomes are shown in Fig. 2 and Additional file 4: Table S2. The risks for most of adverse maternal and neonatal outcomes increased significantly with increased maternal age, while the risk for PPH among age groups did not differ. In addition, the risks for placental previa as well as placental abruption increased at maternal age ≥ 35 years, but did not further increase at maternal age ≥ 43 years, when the risk of maternal ICU admission and neonatal death at birth began to elevate.

**Fig. 3:**
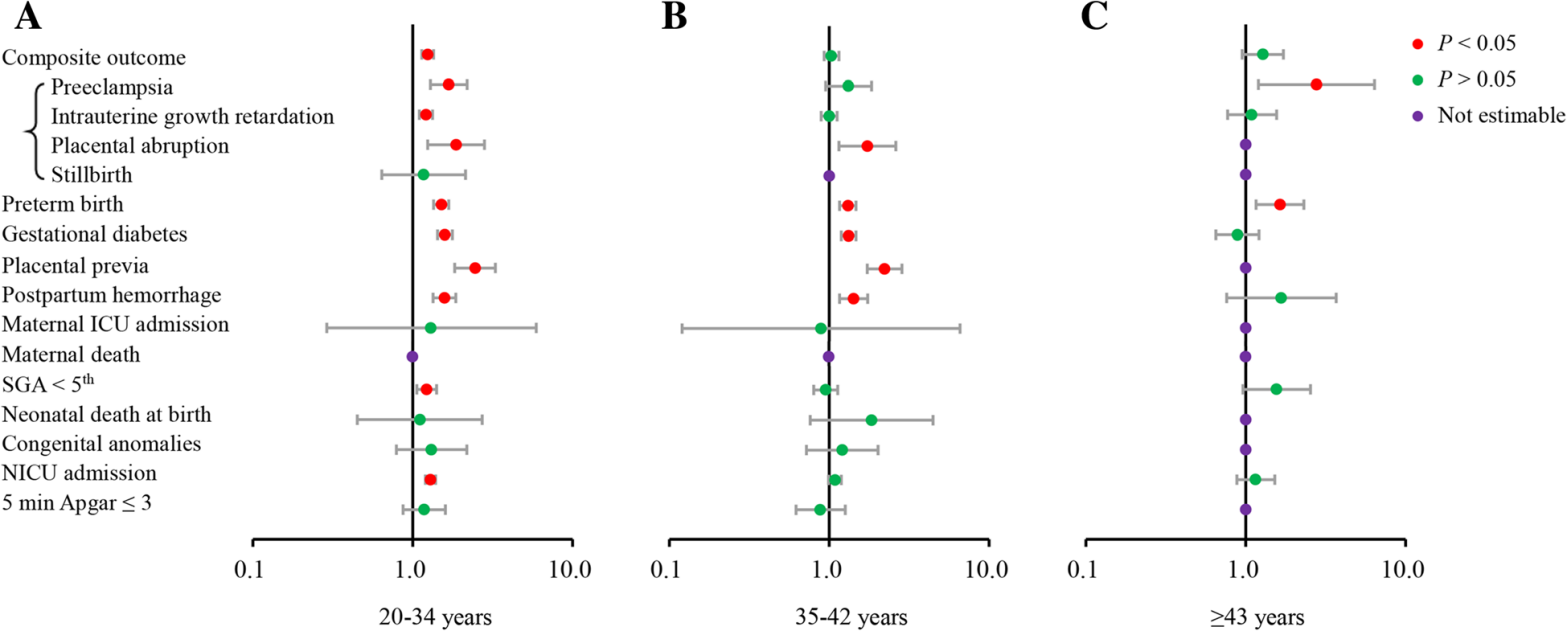
Effect size of type of conception (assisted reproductive technology vs. spontaneous conception) on adverse maternal and neonatal outcomes, stratified by maternal age. The composite outcome includes preeclampsia, intrauterine growth retardation, placental abruption and stillbirth. Models for maternal outcomes were adjusted for parity, neighborhood income, educational level, pre-pregnancy body mass index, drug/alcohol/tobacco use, maternal pre-existing health problems (preexisting hypertension, pre-existing diabetes mellitus, maternal heart disease, maternal pulmonary diseases, maternal endocrine disorders, hematologic disorders). Models for neonatal outcomes were adjusted for parity, neighborhood income, educational level, pre-pregnancy body mass index, drug/alcohol/tobacco use, maternal pre-existing health problems, gestational diabetes mellitus, and preeclampsia. Dots signify relative risks, and bars signify 95% confidence intervals. Red dots signify significant relative risks, green dots signify insignificant results, and purple dots signify non-estimable results

ARRs and 95% CIs for maternal and neonatal outcomes between ART versus SC in different maternal age groups are presented in Fig. 3 and Additional file 5: Table S3. The risk of the primary outcome was higher among ART pregnancies compared to SC for women aged 20–34 years, but not for women aged 35–42 years and 43 years or older. Similarly, for secondary outcomes, higher risks for IUGR, SGA <5th percentile, and NICU admission in ART than SC were only observed in women aged 20–34 years. In contrast, the risk of preeclampsia and preterm birth were higher in ART in women aged ≥43 years. The risk of maternal ICU admission, neonatal death at birth, sentinel congenital anomalies, and 5 min Apgar score ≤ 3 did not differ between methods of conception across all age groups. No significant interactions were identified between vAMA and ART, parity, education, BMI, drugs/alcohol/smoking and pre-existing health problem in this study (data not shown).

Associations between maternal age and adverse maternal and neonatal outcomes, stratified by method of conception are presented in Additional file 6: Table S4. The risks of adverse pregnancy outcomes with advanced age did not differ substantially between women with SC and women in general. However, for women conceived with ART, the effect sizes of maternal age on pregnancy outcomes were generally smaller. The risk for primary outcome significantly increased in women aged 43 years or older compared to that in women under 43, also did the risks for preeclampsia, and preterm birth. Besides, in addition to PPH, the risk of IUGR, placental abruption, NICU admission, and 5 min Apgar score ≤ 3 did not increase with advanced maternal age.

## Discussion

Our large population-based study in Ontario found that mothers at vAMA had a higher risk of developing an adverse composite outcome consisting of preeclampsia, intrauterine growth retardation, stillbirth, and placental abruption, compared to younger counterparts. Increased risk of the composite outcome in ART pregnancies was only observed in women aged 20–34 years, compared to spontaneous conception. For women conceived by ART, the risk for the adverse composite outcome significantly increased in women at vAMA than that in younger women.

There is an increasing prevalence of women who tended to delay childbirth for various reasons worldwide [4–6]. In 2016, the birth rate for women aged ≥45 was 0.9 per 1000 women, the highest rate for this age group since 1963 [24]. As the number of vAMA women increases, the impact of vAMA on birth outcomes remains underappreciated. A recent retrospective cohort study examined the risk for severe maternal morbidity and pregnancy complications across maternal age during delivery hospitalizations in the U.S. between 2006 and 2015 [25]. The authors analyzed a total of 36,944,292 deliveries, and concluded that women aged ≥45 years old were at the highest risk for a broad range of adverse outcomes during delivery hospitalizations among all age groups, with an ARR of 3.46 (95% CI: 3.15–3.80) for severe maternal morbidity compared women aged 25–29. The effect size was larger than that in our study, possibly owing the differences in the definition of outcome and the reference group.

There has been a significantly increased use of ART for women at AMA, as fertility progressively decreases after age 35 years [3, 26]. It is known that ART carry its own increased risks of adverse pregnancy outcomes [1, 18, 19]. Although the further increased risk should be considered for women of advanced maternal age considering pregnancy with ART, all pregnancy outcomes analyzed in this study, except preeclampsia and preterm birth, were not statistically different between in ART and in SC pregnancy among women at vAMA. The effect of ART on pregnancy outcomes seemed to be stronger in younger women, since that a higher risk of ischemic placental diseases in ART than SC was only observed in women aged 20–34 years; this is consistent with previous reports [1, 19]. When further analysis was performed with stratification by the method of conception, we found that ART did not synergistically enhance the effect of vAMA on adverse pregnancy outcomes. The findings may be due to several reasons. First, as the economy of Ontario, Canada thrives, its medical technology industry becomes vibrant, diverse, and expanding. Women in Ontario, especially those at vAMA, will undergo prenatal screening before receiving ART; this may be a primary reason for good pregnancy outcomes. Second, women at vAMA who conceived through ART have higher socioeconomic status than compared to those conceived spontaneously; this may mitigate the adverse effect of vAMA on pregnancy outcomes [27]. Furthermore, the indications for ART may be different in older and younger women [19]. The use of ART in older women tends to be a result of age-related infertility, while younger women who receive ART are more likely to have pre-existing diseases that may contribute to the increased risks in pregnancy. As a result, women at AMA or even vAMA under good health condition and the supervision of qualified medical professionals may have similar pregnancy outcomes compared to young women.

The strengths of this study include a large sample size with the most recent data from Canada, and a population-based design. Although several prior studies of vAMA patients recognized increased risks of adverse pregnancy outcomes and outcomes associated with fertility treatment, [1, 18, 28, 29] few have specially analyzed the increased risks for ischemic placental diseases in women at vAMA and addressed the interplay between age-related and ART-related risks.

Despite a large study population, there are limitations in our study. First, we only investigated the short-term neonatal outcomes. Recently, researchers have linked an individual’s susceptibility to chronic disease, such as cardiovascular disease, diabetes, and obesity in adult life to events during their intrauterine phase of development [30]. Therefore, further studies on long-term outcomes would help provide epidemiologic evidence regarding the associations between chronic diseases and events during their intrauterine phase. Second, missing data or incomplete ascertainment of certain outcomes were observed in our study, which may lead to a biased estimation of the effect size [31].

## Conclusion

Women at vAMA have increased risk of adverse pregnancy outcomes. Although ART is an independent risk for adverse outcomes, it does not further enhance the effect of vAMA. Regardless of the method of conception, for women conceive at vAMA, the needs for preconception counseling, greater antenatal care and better management, such as targeted surveillance and early intervention [10] should be met.

## Additional files

Additional file 1: **S1**. Sentinel Congenital Anomaly BIS Pick List and CIHI ICD-10-CA Mapping. (PDF 89 kb)

Additional file 2: **Figure S1**. The association of continuous maternal age with rate of the primary outcome (preeclampsia, intrauterine growth retardation, placental abruption and stillbirth). (A) Overall. (B) Stratified by the method of conception. (DOCX 35 kb)

Additional file 3: **Table S1**. Incidence of adverse maternal and neonatal outcomes among maternal age groups, stratified by the method of conception. (DOCX 20 kb)

Additional file 4: **Table S2**. Association of advanced and very advanced maternal age with adverse maternal and neonatal outcomes. (DOCX 19 kb)

Additional file 5: **Table S3**. Association of assisted reproductive technology with adverse maternal and neonatal outcomes, stratified by maternal age. (DOCX 19 kb)

Additional file 6: **Table S4**. Association of advanced and very advanced maternal age with adverse maternal and neonatal outcomes, stratified by the method of conception. (DOCX 21 kb)

## Abbreviations

AMA: advanced maternal age
ARR: adjusted relative risks
ART: assisted reproductive technology
BMI: body mass index
BORN: Better Outcomes Registry & Network
GDM: gestational diabetes mellitus
ICSI: intracytoplasmic sperm injection
IQR: interquartile range
IUGR: intrauterine growth retardation
IUI: intrauterine insemination
IVF: in-vitro fertilization
NICU: neonatal intensive care unit
PPH: postpartum hemorrhage
SC: spontaneous conception
SGA: small for gestational age
vAMA: very advanced maternal age
