# Supplementary figures and images for: Adverse maternal and neonatal outcomes among singleton pregnancies in women of very advanced maternal age: a retrospective cohort study
Source: BMC Pregnancy Childbirth. 2019 Jan 3;19:3. doi: 10.1186/s12884-018-2147-9 (PMC6318893; doi:10.1186/s12884-018-2147-9)

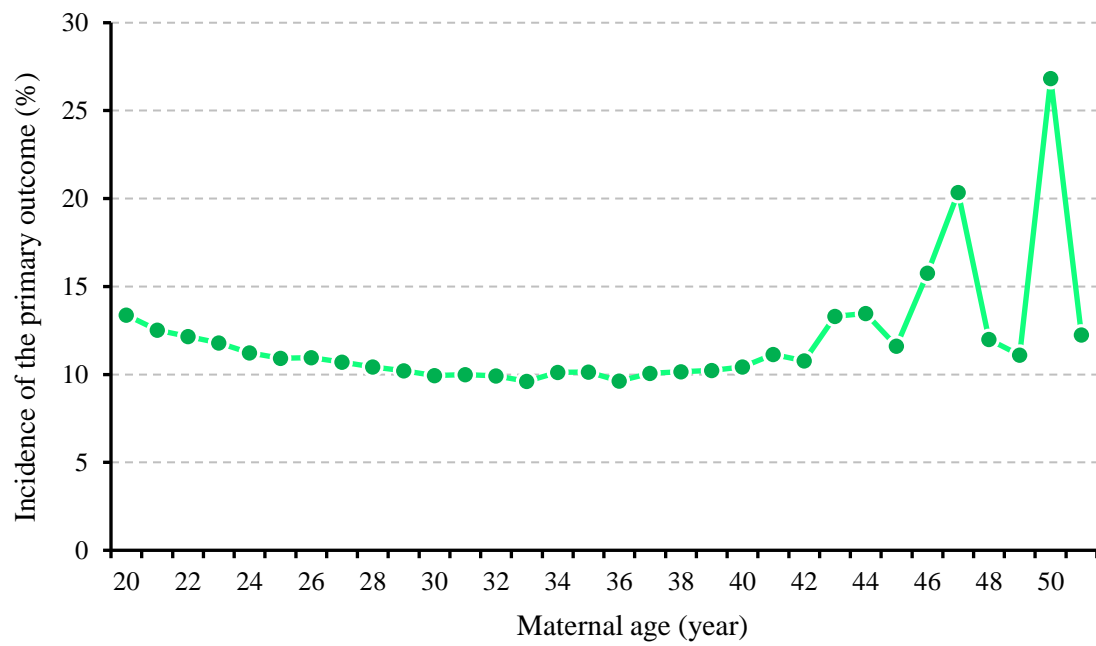

(A)

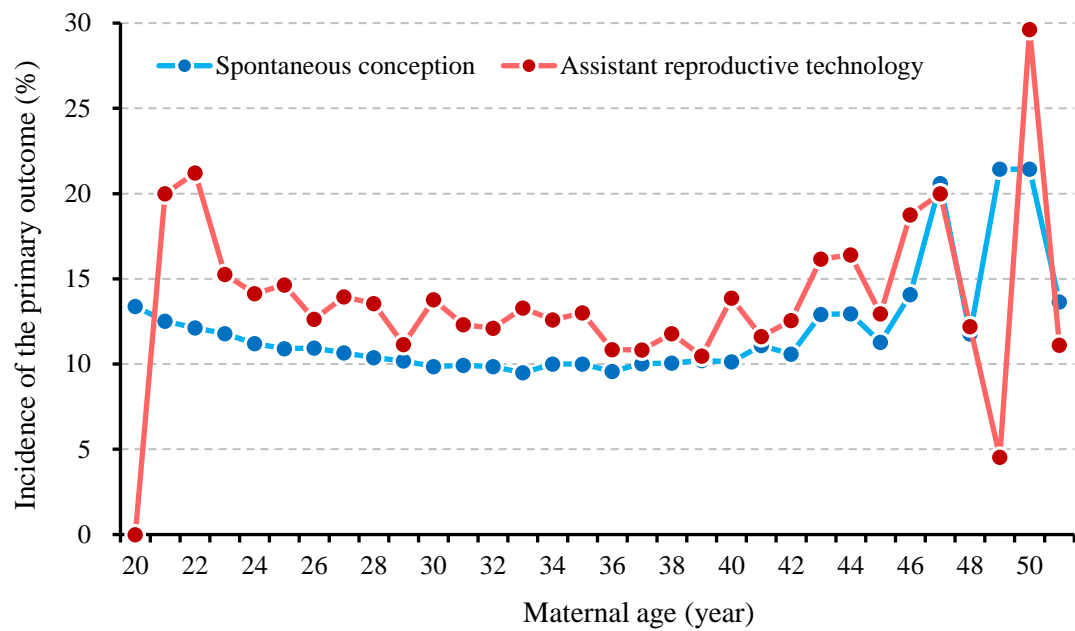

(B)

Supplement: Supplementary file 1 — S1. Sentinel Congenital Anomaly BIS Pick List and CIHI ICD-10-CA Mapping. (PDF 89 kb) [file 12884_2018_2147_MOESM1_ESM.pdf]
