# Supplementary material for: Adverse maternal and neonatal outcomes among singleton pregnancies in women of very advanced maternal age: a retrospective cohort study
Source: BMC Pregnancy Childbirth. 2019 Jan 3;19:3. doi: 10.1186/s12884-018-2147-9 (PMC6318893; doi:10.1186/s12884-018-2147-9)
Supplement: Supplementary file 2 — Figure S1. The association of continuous maternal age with rate of the primary outcome (preeclampsia, intrauterine growth retardation, placental abruption and stillbirth). (A) Overall. (B) Stratified by the method of conception. (DOCX 35 kb) [file 12884_2018_2147_MOESM2_ESM.docx]

Sentinel congenital anomalies include following newborn conditions: neural tube defects, anencephaly, Spina Bifida, hydrocephalus (without spina bifida (excl. hydranenecephaly)), Cleft Lip +/- Cleft palate, cleft palate, esophageal atresia/stenosis, Tracheooesphageal Fistula, small & large intestinal atresia/stenosis, hypospadias/epispadias (male only), limb reductions, gastroschisis, omphalocele, renal agenesis, Hypoplastic Left Heart Syndrome, Tetralogy of Fallot (TOF), Transposition of great vessels (TGA), Down Syndrome, Trisomy 18 and Trisomy 13.

|  | BIS *(mapping as per pick list value names)* | CIHI *(ICD-10, ICD-10-CA)* |
| --- | --- | --- |
| Neural Tube  Defects (all) | - Head-Cranium & Brain \ Anencephaly - Head-Cranium & Brain \ Acrania - Head-Cranium & Brain \ Craniorachischisis - Head-Cranium & Brain\| Encephalocele - Head-Cranium & Brain \ Hydranencephaly - Spine - Back \ NTD with hydrocephalus - Spine - Back \ NTD without hydrocephalus | **Q00.., Q01.., Q05..**   - Q00.^^:Anencephaly and similar malformations   - Q01.^^: Encephalocele, encephalomyelocele, hydroencephalocele, hydromeningocele, cranial, meningocele, cerebral, meningoencephalocele - Q05.^^:Spina bifida |
| Anencephaly | - Head-Cranium & Brain \ Anencephaly - Head-Cranium & Brain \ Acrania - Head-Cranium & Brain \ Craniorachischisis | Q00.00, Q00.01, Q00.1   - Q00.0: Anencephaly   - Acephaly, acrania, amyelencephaly, hemianencephaly, hemicephaly - Q00.1: Craniorachischisis |
| SpinaBifida(SB) | •Spine - Back \ NTD with hydrocephalus  •Spine - Back \ NTD without hydrocephalus  **Naming is NTD in the BIS, notSpina Bifida (SB).* | Q05..   - Q05.^^:Spina bifida   - hydromeningocele (spine), meningocele (spinal), meningomyelocele, myelocele, myelomeningocele, rachischisis, spina bifida (aperta)(cystica), syringomyelocele |
| Hydrocephalus (without Spina Bifida (excl. hydranenecephaly)) | - Head-Cranium & Brain \ Hydrocephalus - Head-Cranium & Brain \ Atresia of foramina of Magendie&Luschka - Head-Cranium & Brain \ Dandy-Walker malformation / variant (DWM) - Syndromes \ Dandy-walker syndrome   •Head-Cranium & Brain \ Aqueductal stenosis  •Head-Cranium & Brain \ Hydrochephalus X-Linked | Q03   - Q03.^^: Congenital hydrocephalus   - malformations of aqueduct of Sylvius   - Atresia of foramina of Magendie and Luschka, Dandy-Walker syndrome |
| Cleft Lip +/- Cleft Palate | - Face \ MOUTH-Cleft lip & palate - Face \ MOUTH-Cleft lip | Q36.., Q37..   - Q36.^^: Cleft lip (excludes lip w/ palate) - Q37.^^: Cleft palate with cleft lip |
| Cleft Palate | - Face \ MOUTH-Cleft palate | Q35..   - Q35.^^: Cleft palate |
| **Oesophageal**  **Atresia/Stenosis**  Tracheo-oesphageal Fistula | - Gastrointestinal \ Esophagealatresia - Gastrointestinal \ Tracheoesophagealfistula (TEF) | Q39.0 – Q39.4   - Q39.0: Atresia of oesophagus without fistula - Q39.1: Atresia of oesophagus with tracheo-oesophageal fistula (includes w/ bronchi-EF) - Q39.2: Congenital traceho-oesophageal fistula without atresia - Q39.3: Congenital stenosis and stricture of oesophagus - Q39.4: Oesophageal web |
| Small & Large Intestinal Atresia/Stenosis | • Gastrointestinal \ Atresia small or large intestine  • Gastrointestinal \ Duodenal atresia  • Gastrointestinal \ Abnormal small or large Bowel  •Gastrointestinal \ Double bubble  • Gastrointestinal \ Bowel obstruction sm/lgintestine  • Gastrointestinal \ Stenois small or large intestine  • Gastrointestinal \ Imperforate anus  ** Cannot separate sm&lgintestine atresia in BIS.* | Q41..   - Q41.^^: Congenital absence, atresia and stenosis of small intestine   Q42..   - Q42.^^: Congenital absence, atresia and stenosis of large intestine   - congenital obstruction, occlusion and stricture of large intestine   - imperforate rectum, imperforate anus, large intestine, anus, or rectum |
| Hypospadias/Epispadias (Male only) | - Genitourinary Tract \ Hypospadias   ** BIS does not collect epispadias.* | Q54 (excl. Q54.4), Q64.0   - Q54.^^: Hypospadias   - penile, penoscrotal, perineal, other, unspecified   - EXCLUDE Q54.4: Congenital chordee - Q64.0: Epispadias |
| Limb Reductions | - Extremities-skeletal \ Generalized/other-Limb reduction defect(s) (LRD) - lower limb - Extremities-skeletal \ Generalized/other-Limb reduction defect(s) (LRD) - upper limb   •Extremities-skeletal \ Hands/feet-Radial ray anomaly (absent thumb)  •Extremities-skeletal \ Hands/feet-Adactyly (absent fingers/ toes)  •Extremities-skeletal \ Hands/feet-Ectrodactyly (lobster-claw / cleft hand | Q72.. (lower) Q71.. (upper)   - Q72.^^: Reduction defects of lower limb - Q71.^^: Reduction defects of upper limb |
| Gastroschisis | - Abdominal Wall \ Gastroschisis | Q79.3   - Q79.3: Gastroschisis |
| Omphalocele | - Abdominal Wall \ Omphalocele (exomphalos) | Q79.2   - Q79.2: Exomphalos (includes Omphalocele, excludes umbilical hernia) |
| Renal Agenesis | - Genitourinary Tract \ Renal agenesis | Q60..   - Q60.^^: Renal agenesis and other reduction defects of kidney   - atrophy of kidney   - congenital absence of kidney |
| **Hypoplastic Left**  Heart Syndrome | - Cardiovascular \ Hypoplastic left heart syndrome (HLHS) | Q23.4   - Q23.4: Hypoplastic left heart syndrome   - atresia, or marked hypoplasia of aortic orifice or valve, with hypoplasia of ascending aorta and defective development of left ventricle (with mitral valve stenosis or atresia) |
| **Tetralogy of Fallot (TOF)** | • Cardiovascular \ Tetralogy of Fallot (TOF) | Q21.3  • Q21.3: Tetralogy of Fallot |
| **Transposition of great vessels (TGA)** | • Cardiovascular \ Transposition of great vessels (TGA)  * and includes by default:  • Cardiovascular \ Transposition of great arteries - congenitally corrected (CCTGA) | Q20.3  • Q20.3: Discordant ventriculoarterial connection (includes Dextrotransposition of aorta, Transposition of great vessels (complete)). |
| Down Syndrome | - Chromosomes \ Trisomy 21 (Down syndrome) - Chromosomes \ Trisomy 21 (Down syndrome) - translocation - Chromosomes \ Trisomy 21 (Down syndrome) - mosaic | Q90..   - Q90.^^: Down’s Syndrome (meiotic nondisjunction, translocation, unspecified) |
| Trisomy 18 | - Chromosomes \ Trisomy 18 | Q91.0-Q91.3   - Q91.0: Trisomy 18, meiotic nondisjunction - Q91.1: Trisomy 18, mosaicism (mitotic nondisjunction) - Q91.2: Trisomy 18, nondisjunction - Q91.3: Edwards’ syndrome, unspecified |
| Trisomy 13 | - Chromosomes \ Trisomy 13 | Q91.4-Q91.7   - Q91.4: Trisomy 13, meiotic nondisjunction - Q91.5: Trisomy 13, mosaicism (mitotic nondisjunction) - Q91.6: Trisomy 13, translocation - Q91.7: Patau’s syndrome, unspecified |
