# Supplementary material for: Adverse maternal and neonatal outcomes among singleton pregnancies in women of very advanced maternal age: a retrospective cohort study
Source: BMC Pregnancy Childbirth. 2019 Jan 3;19:3. doi: 10.1186/s12884-018-2147-9 (PMC6318893; doi:10.1186/s12884-018-2147-9)
Supplement: Supplementary file 3 — Table S1. Incidence of adverse maternal and neonatal outcomes among maternal age groups, stratified by the method of conception. (DOCX 20 kb) [file 12884_2018_2147_MOESM3_ESM.docx]

**Table S1. Incidence of adverse maternal and neonatal outcomes among maternal age groups, stratified by the method of conception**

|  | Spontaneous conception | | |  | Assisted reproductive technology | | |
| --- | --- | --- | --- | --- | --- | --- | --- |
| Outcome | **20-34 years** | **35-42 years** | **≥ 43 years** |  | **20-34 years** | **35-42 years** | **≥ 43 years** |
| N | 292,666 | 78,925 | 2,608 |  | 6,178 | 4,988 | 658 |
| Composite outcome (preeclampsia, IUGR, placental abruption and stillbirth) | 30,307 (10.36) | 7,876 (9.98) | 330 (12.65) |  | 795 (12.87) | 587 (11.77) | 106 (16.11) |
| Preeclampsia | 2,111 (0.72) | 629 (0.80) | 27 (1.04) |  | 104 (1.68) | 70 (1.40) | 25 (3.80) |
| IUGR | 26,949 (9.21) | 6,833 (8.66) | 281 (10.77) |  | 667 (10.80) | 481 (9.65) | 77 (11.70) |
| Placental abruption | 1,282 (0.44) | 448 (0.57) | 20 (0.77) |  | 48 (0.78) | 47 (0.94) | 10 (1.52) |
| Stillbirth | 801 (0.27) | 265 (0.34) | 21 (0.81) |  | 21 (0.34) | 25 (0.50) | < 6 |
| Preterm birth | 16,670 (5.70) | 5,404 (6.85) | 221 (8.47) |  | 529 (8.56) | 466 (9.34) | 94 (14.29) |
| Gestational diabetes mellitus | 13,111 (4.48) | 6,864 (8.7) | 371 (14.23) |  | 507 (8.21) | 529 (10.61) | 85 (12.92) |
| Placental previa | 1,584 (0.54) | 826 (1.05) | 31 (1.19) |  | 83 (1.34) | 114 (2.29) | 24 (3.65) |
| Postpartum hemorrhage | 7,292 (2.49) | 1,669 (2.11) | 57 (2.19) |  | 241 (3.90) | 167 (3.35) | 19 (2.89) |
| Maternal ICU admission | 90 (0.03) | 40 (0.05) | <6 |  | < 6 | < 6 | < 6 |
| Maternal death related to pregnancy and birth | < 6 | < 6 | < 6 |  | 0 (0.0) | 0 (0.0) | 0 (0.0) |
| SGA < 5^th^ | 12,413 (4.24) | 3,219 (4.08) | 129 (4.95) |  | 323 (5.23) | 221 (4.43) | 40 (6.08) |
| Neonatal death | 395 (0.13) | 106 (0.13) | 7 (0.27) |  | 16 (0.26) | 9 (0.18) | < 6 |
| Sentinel congenital anomalies | 975 (0.33) | 336 (0.43) | 37 (1.42) |  | 30 (0.49) | 24 (0.48) | < 6 |
| NICU admission | 34,111 (11.66) | 9,686 (12.27) | 392 (15.03) |  | 985 (15.94) | 737 (14.78) | 126 (19.15) |
| 5 min Apgar ≤ 3 | 3,017 (1.03) | 943 (1.19) | 52 (1.99) |  | 83 (1.34) | 65 (1.30) | 8 (1.22) |

IUGR: intrauterine growth retardation. ICU: intensive care unit. SGA: small for gestational age. NICU: neonatal intensive care unit. N/A: not applicable.
