# Supplementary material for: Adverse maternal and neonatal outcomes among singleton pregnancies in women of very advanced maternal age: a retrospective cohort study
Source: BMC Pregnancy Childbirth. 2019 Jan 3;19:3. doi: 10.1186/s12884-018-2147-9 (PMC6318893; doi:10.1186/s12884-018-2147-9)
Supplement: Supplementary file 4 — Table S2. Association of advanced and very advanced maternal age with adverse maternal and neonatal outcomes. (DOCX 19 kb) [file 12884_2018_2147_MOESM4_ESM.docx]

**Table S2. Association of advanced and very advanced maternal age with adverse maternal and neonatal outcomes ^a^**

| Outcome | ≥ 43 years vs. 20-34 years | 35-42 years vs. 20-34 years | ≥ 43 years vs. 35-42 years |
| --- | --- | --- | --- |
|  | **ARR (95% CI)** | **ARR (95% CI)** | **ARR (95% CI)** |
| Composite outcome (preeclampsia, IUGR, placental abruption and stillbirth) | 1.38 (1.23, 1.55) | 1.10 (1.06, 1.13) | 1.26 (1.12, 1.42) |
| Preeclampsia | 2.32 (1.63, 3.30) | 1.18 (1.04, 1.33) | 1.97 (1.37, 2.82) |
| IUGR | 1.26 (1.10, 1.44) | 1.08 (1.05, 1.12) | 1.17 (1.02, 1.34) |
| Placental abruption | 2.09 (1.28, 3.40) | 1.42 (1.23, 1.65) | 1.47 (0.90, 2.39) |
| Stillbirth | 2.80 (1.58, 4.97) | 1.27 (1.04, 1.55) | 2.21 (1.24, 3.94) |
| Preterm birth | 1.53 (1.33, 1.76) | 1.19 (1.14, 1.24) | 1.29 (1.12, 1.49) |
| Gestational diabetes mellitus | 2.28 (2.04, 2.56) | 1.62 (1.56, 1.68) | 1.41 (1.26, 1.58) |
| Placental Previa | 2.42 (1.70, 3.45) | 1.77 (1.58, 1.98) | 1.37 (0.96, 1.95) |
| Postpartum hemorrhage | 0.88 (0.64, 1.20) | 0.98 (0.91, 1.05) | 0.90 (0.65, 1.23) |
| Maternal ICU admission | 6.88 (2.29, 20.62) | 1.57 (0.93, 2.67) | 4.37 (1.46, 13.1) |
| Maternal death related to pregnancy and birth | N/A | N/A | N/A |
| SGA < 5^th^ | 1.42 (1.16, 1.72) | 1.15 (1.09, 1.21) | 1.24 (1.01, 1.51) |
| Neonatal death | 2.53 (1.20, 5.34) | 0.94 (0.70, 1.26) | 2.69 (1.25, 5.81) |
| Sentinel Congenital Anomalies | 2.89 (1.73, 4.84) | 1.23 (1.03, 1.48) | 2.34 (1.40, 3.91) |
| NICU admission | 1.26 (1.13, 1.40) | 1.12 (1.09, 1.16) | 1.12 (1.00, 1.25) |
| 5 min Apgar ≤ 3 | 1.96 (1.43, 2.69) | 1.18 (1.07, 1.30) | 1.66 (1.21, 2.28) |

IUGR: intrauterine growth retardation. ICU: intensive care unit. SGA: small for gestational age. NICU: neonatal intensive care unit. N/A: not applicable. ARR: adjusted relative risk.

^a^ Models for maternal outcomes were adjusted for parity, neighborhood income, educational level, pre-pregnancy body mass index, drug/alcohol/tobacco use, type of conception, maternal pre-existing health problems (preexisting hypertension, pre-existing diabetes mellitus, maternal heart disease, maternal pulmonary diseases, maternal endocrine disorders, hematologic disorders). Models for neonatal outcomes were adjusted for parity, neighborhood income, educational level, pre-pregnancy body mass index, drug/alcohol/tobacco use, type of conception, maternal pre-existing health problems, gestational diabetes mellitus, and preeclampsia.
