# Supplementary material for: Adverse maternal and neonatal outcomes among singleton pregnancies in women of very advanced maternal age: a retrospective cohort study
Source: BMC Pregnancy Childbirth. 2019 Jan 3;19:3. doi: 10.1186/s12884-018-2147-9 (PMC6318893; doi:10.1186/s12884-018-2147-9)
Supplement: Supplementary file 5 — Table S3. Association of assisted reproductive technology with adverse maternal and neonatal outcomes, stratified by maternal age. (DOCX 19 kb) [file 12884_2018_2147_MOESM5_ESM.docx]

**Table S3. Association of assisted reproductive technology with adverse maternal and neonatal outcomes, stratified by maternal age group ^a^**

| Outcome | 20-34 years | 35-42 years | ≥ 43 years |
| --- | --- | --- | --- |
|  | **ARR (95% CI)** | **ARR (95% CI)** | **ARR (95% CI)** |
| Composite outcome (preeclampsia, IUGR, placental abruption and stillbirth) | 1.24 (1.14, 1.35) | 1.03 (0.93, 1.15) | 1.28 (0.95, 1.72) |
| Preeclampsia | 1.68 (1.29, 2.19) | 1.32 (0.95, 1.84) | 2.77 (1.20, 6.39) |
| IUGR | 1.21 (1.10, 1.33) | 1.00 (0.89, 1.12) | 1.09 (0.77, 1.56) |
| Placental abruption | 1.87 (1.24, 2.81) | 1.73 (1.15, 2.61) | N/A |
| Stillbirth | 1.17 (0.64, 2.14) | N/A | N/A |
| Preterm birth | 1.51 (1.35, 1.68) | 1.31 (1.16, 1.47) | 1.64 (1.16, 2.31) |
| Gestational diabetes mellitus | 1.59 (1.43, 1.77) | 1.32 (1.19, 1.47) | 0.89 (0.65, 1.21) |
| Placental Previa | 2.46 (1.83, 3.29) | 2.22 (1.73, 2.85) | N/A |
| Postpartum hemorrhage | 1.58 (1.34, 1.86) | 1.42 (1.16, 1.74) | 1.67 (0.76, 3.68) |
| Maternal ICU admission | 1.30 (0.29, 5.93) | 0.89 (0.12, 6.58) | N/A |
| Maternal death related to pregnancy and birth | N/A | N/A | N/A |
| SGA < 5^th^ | 1.22 (1.06, 1.41) | 0.95 (0.80, 1.13) | 1.56 (0.96, 2.54) |
| Neonatal death | 1.11 (0.45, 2.72) | 1.84 (0.76, 4.46) | N/A |
| Sentinel Congenital Anomalies | 1.31 (0.79, 2.18) | 1.21 (0.72, 2.02) | N/A |
| NICU admission | 1.29 (1.20, 1.39) | 1.09 (0.99, 1.19) | 1.15 (0.88, 1.52) |
| 5 min Apgar ≤ 3 | 1.18 (0.87, 1.60) | 0.88 (0.62, 1.26) | N/A |

IUGR: intrauterine growth retardation. ICU: intensive care unit. SGA: small for gestational age. NICU: neonatal intensive care unit. N/A: not applicable. ARR: adjusted relative risk.

^a^ Models for maternal outcomes were adjusted for parity, neighborhood income, educational level, pre-pregnancy body mass index, drug/alcohol/tobacco use, maternal pre-existing health problems (preexisting hypertension, pre-existing diabetes mellitus, maternal heart disease maternal pulmonary diseases, maternal endocrine disorders, hematologic disorders). Models for neonatal outcomes were adjusted for parity, neighborhood income, educational level, pre-pregnancy body mass index, drug/alcohol/tobacco use, maternal pre-existing health problems, gestational diabetes mellitus, and preeclampsia.
