# Supplementary material for: Adverse maternal and neonatal outcomes among singleton pregnancies in women of very advanced maternal age: a retrospective cohort study
Source: BMC Pregnancy Childbirth. 2019 Jan 3;19:3. doi: 10.1186/s12884-018-2147-9 (PMC6318893; doi:10.1186/s12884-018-2147-9)
Supplement: Supplementary file 6 — Table S4. Association of advanced and very advanced maternal age with adverse maternal and neonatal outcomes, stratified by the method of conception. (DOCX 21 kb) [file 12884_2018_2147_MOESM6_ESM.docx]

**Table S4. Association of advanced and very advanced maternal age with adverse maternal and neonatal outcomes, stratified by type of conception ^a^**

| Outcome | ≥ 43 years vs. 20-34 years | 35-42 years vs. 20-34 years | ≥ 43 years vs. 35-42 years |
| --- | --- | --- | --- |
|  | **ARR (95% CI)** | **ARR (95% CI)** | **ARR (95% CI)** |
| Assisted reproductive technology |  |  |  |
| Composite outcome (preeclampsia, IUGR, placental abruption and stillbirth) | 1.29 (1.01, 1.65) | 0.95 (0.83, 1.08) | 1.36 (1.06, 1.74) |
| Preeclampsia | 3.21 (1.90, 5.45) | 0.90 (0.60, 1.35) | 3.57 (2.07, 6.18) |
| IUGR | 1.08 (0.81, 1.45) | 0.93 (0.81, 1.08) | 1.16 (0.86, 1.56) |
| Placental abruption | 1.36 (0.48, 3.86) | 1.23 (0.71, 2.13) | 1.11 (0.39, 3.13) |
| Stillbirth | N/A | N/A | N/A |
| Preterm birth | 1.50 (1.14, 1.97) | 1.02 (0.87, 1.19) | 1.47 (1.12, 1.94) |
| Gestational diabetes mellitus | 1.42 (1.06, 1.91) | 1.36 (1.17, 1.57) | 1.05 (0.79, 1.40) |
| Placental previa | 1.87 (0.93, 3.76) | 1.77 (1.21, 2.58) | 1.06 (0.55, 2.05) |
| Postpartum hemorrhage | 0.75 (0.43, 1.32) | 0.81 (0.63, 1.04) | 0.92 (0.52, 1.64) |
| Maternal ICU admission | N/A | N/A | N/A |
| Maternal death related to pregnancy and birth | N/A | N/A | N/A |
| SGA< 5^th^ | 1.37 (0.92, 2.05) | 0.90 (0.72, 1.13) | 1.52 (1.01, 2.28) |
| Neonatal death | N/A | N/A | N/A |
| Sentinel congenital anomalies | N/A | N/A | N/A |
| NICU admission | 1.10 (0.87, 1.38) | 0.91 (0.82, 1.02) | 1.20 (0.95, 1.51) |
| 5 min Apgar ≤ 3 | 1.01 (0.40, 2.55) | 0.92 (0.58, 1.45) | 1.11 (0.43, 2.82) |
| Spontaneous conception |  |  |  |
| Composite outcome (preeclampsia, IUGR, placental abruption and stillbirth) | 1.37 (1.20, 1.57) | 1.11 (1.07, 1.14) | 1.24 (1.08, 1.42) |
| Preeclampsia | 1.67 (0.99, 2.83) | 1.21 (1.07, 1.37) | 1.38 (0.81, 2.35) |
| IUGR | 1.29 (1.11, 1.50) | 1.09 (1.05, 1.13) | 1.19 (1.02, 1.38) |
| Placental abruption | 2.3 (1.33, 3.98) | 1.45 (1.24, 1.69) | 1.59 (0.91, 2.76) |
| Stillbirth | 3.31 (1.85, 5.92) | 1.27 (1.03, 1.56) | 2.61 (1.45, 4.70) |
| Preterm birth | 1.49 (1.27, 1.76) | 1.20 (1.15, 1.25) | 1.24 (1.05, 1.47) |
| Gestational diabetes mellitus | 2.49 (2.20, 2.81) | 1.63 (1.57, 1.70) | 1.52 (1.35, 1.72) |
| Placental previa | 2.66 (1.76, 4.02) | 1.76 (1.56, 1.98) | 1.51 (1.00, 2.29) |
| Postpartum hemorrhage | 0.87 (0.59, 1.28) | 1.00 (0.93, 1.07) | 0.87 (0.59, 1.29) |
| Maternal ICU admission | 6.88 (2.02, 23.45) | 1.68 (0.99, 2.83) | 4.10 (1.19, 14.19) |
| Maternal death related to pregnancy and birth | N/A | N/A | N/A |
| SGA < 5^th^ | 1.38 (1.10, 1.73) | 1.16 (1.10, 1.22) | 1.19 (0.94, 1.50) |
| Neonatal death | 1.47 (0.47, 4.64) | 0.91 (0.67, 1.23) | 1.62 (0.51, 5.18) |
| Sentinel congenital anomalies | 3.77 (2.29, 6.22) | 1.23 (1.02, 1.48) | 3.06 (1.85, 5.06) |
| NICU admission | 1.27 (1.13, 1.44) | 1.14 (1.10, 1.17) | 1.12 (0.99, 1.27) |
| 5 min Apgar ≤ 3 | 2.18 (1.56, 3.03) | 1.19 (1.08, 1.32) | 1.83 (1.31, 2.55) |

IUGR: intrauterine growth retardation. ICU: intensive care unit. SGA: small for gestational age. NICU: neonatal intensive care unit. N/A: not applicable. ARR: adjusted relative risk.

^a^ Models for maternal outcomes were adjusted for parity, neighborhood income, educational level, pre-pregnancy body mass index, drug/alcohol/tobacco use, maternal pre-existing health problems (preexisting hypertension, pre-existing diabetes mellitus, maternal heart disease maternal pulmonary diseases, maternal endocrine disorders, hematologic disorders). Models for neonatal outcomes were adjusted for parity, neighborhood income, educational level, pre-pregnancy body mass index, drug/alcohol/tobacco use, maternal pre-existing health problems, gestational diabetes mellitus, and preeclampsia.
